# CDK8 Inhibition Increases E2F1 Transcriptional Activity and Promotes STAT3-Dependent Suppression of Mcl-1 in Triple-Negative Breast Cancer Cell Line MDA-MB-468

**DOI:** 10.3390/ijms27020897

**Published:** 2026-01-16

**Authors:** Sandra Do, Shengxi Li, Rui Xiong, Jensen M. Spear, Zhixin Lu, William K. Chan, Wade A. Russu

**Affiliations:** Department of Pharmaceutical Sciences, Thomas J. Long School of Pharmacy, University of the Pacific, Stockton, CA 95211, USA; s_do10@u.pacific.edu (S.D.); s_li37@u.pacific.edu (S.L.); aaw3eb@virginia.edu (R.X.); j_spear2@u.pacific.edu (J.M.S.); luzx@mail.uc.edu (Z.L.); wchan@pacific.edu (W.K.C.)

**Keywords:** kinase inhibitor, CDK8, E2F1, STAT3, Mcl-1, triple-negative breast cancer

## Abstract

The targeting of cyclin dependent kinase 8 (CDK8) as a potential strategy for cancer treatment has been of interest since the identification of CDK8 as an oncogene product. In this report, we communicate the results of our continuing investigation into the effects of CDK8 inhibitor on triple-negative breast cancer cell line MDA-MB-468. Here, we demonstrate that inhibition of CDK8 decreases phosphorylation of CDK8 substrates E2 promoter binding factor 1 (E2F1) at serine 375 and signal transducer and activator of transcription 3 (STAT3) at serine 727 in these cells. Additionally, luciferase expression was increased in E2F1-responsive luciferase plasmid-transfected cells. Expression of E2F1 transcription target, the proapoptotic protein p73, was increased, and expression of antiapoptotic protein myeloid cell leukemia sequence 1 (Mcl-1) was decreased in CDK8 inhibitor-treated cells. We also demonstrate that knockdown of STAT3 or disruption of STAT3 function in MDA-MB-468 cells opposes the effects of CDK8 inhibition on Mcl-1. Together, these results suggest that CDK8 inhibitor treatment can modulate the expression of apoptosis-related proteins p73 and Mcl-1 and continues to highlight the potential cooperative effects of E2F1 and STAT3 in the activity of CDK8 inhibitor against MDA-MB-468 triple-negative breast cancer cells.

## 1. Introduction

The cyclin-dependent kinase 8 (CDK8) protein is involved in many cellular processes [1,2]. A major function of this kinase is participation in the regulation of cell cycle progression [3]. CDK8 can regulate gene transcription by phosphorylation of RNA polymerase II (RNAP II) as a part of the mediator complexes MED12 and MED13 [4,5]. In addition to regulating transcription through the mediator complexes, CDK8 can directly phosphorylate and modulate the activity of transcription factors such as E2F1 and STAT family transcription factors [6,7]. Transcriptional activity can also be affected by CDK8 through phosphorylation of histone H3 and modification of chromatin structure [8]. Dysregulation of CDK8 activity has oncogenic potential [9].

In a significant fraction of colon cancers, the CDK8 gene is a target of chromosomal amplification [10]. Knockdown of CDK8 in colon cancer cells with amplified CDK8 decreases proliferation. Furthermore, transfection of NIH3T3 cells with CDK8 causes oncogenic transformation. It was also shown that the kinase function of CDK8 was required for oncogenic transformation of NIH3T3 cells. In the context of colon cancer cells, CDK8 antagonizes the E2F1 transcription factor’s ability to suppress β-catenin [11]. While there is interest in the potential of CDK8 inhibitor development for colon cancer, CDK8 has also become a target of interest for acute myelogenous leukemia (AML), melanoma, prostate cancer, and breast cancer [12,13,14,15].

It has been demonstrated that overexpression of E2F1 in triple-negative breast cancer (TNBC) cell line MDA-MB-468 induces apoptosis; however, a mechanism for this observation has not been determined [16]. We have been using CDK8 inhibition as a method of activating E2F1 in MDA-MB-468 cells to learn how E2F1 promotes apoptosis in this cell line. Previously, we demonstrated that the triple-negative breast cancer cell line MDA-MB-468 exhibited reduced cell viability and active apoptosis after treatment with a CDK8 inhibitor [17]. These effects were accompanied by upregulation of E2F1 protein and increased STAT3 phosphorylation at tyrosine 705. Both knockdown of E2F1 and disruption of STAT3 attenuated the effects of CDK8 inhibition on cell viability, suggesting a mechanistic role for both transcription factors in CDK8 inhibitor-induced cell death. In the previous studies, we used CDK8 inhibitor Q12 (NSC; D-750901, compound **4** in [17,18,19]). Q12 binds to the ATP binding site of CDK8 with a binding dissociation constant of 0.46 µM. Furthermore, Q12 exhibits a selective antiproliferative profile in the NCI 60 tumor cell panel, with the triple-negative breast cancer cell line MDA-MB-468 being the most sensitive (GI50 = 60 nM) [18]. Here, we report our continuing investigation of the effects of CDK8 inhibition on MDA-MB-468 cells.

## 2. Results

### 2.1. Effects of Inhibiting CDK8 on E2F1 and CDK8 Proteins

**Figure 1:**
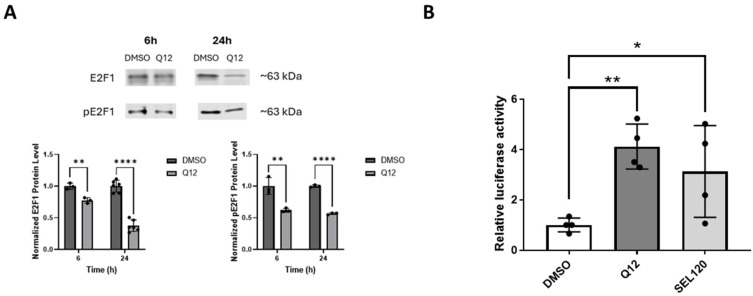
Effect of CDK8 inhibitor Q12 treatment of MDA-MB-468 cells on E2F1 protein and E2F1-dependent luciferase expression. (**A**) Effect of treatment with 10 µM Q12 (6 h and 24 h) on E2F1 (**left**) and pE2F1(Ser375) (**right**) expression in MDA-MB-468 cells compared to vehicle-treated control (DMSO) cells (*n* = 3–6). (**B**) Relative luciferase expression in MDA-MB-468 cells transfected with an E2F1-responsive luciferase plasmid following treatment with Q12 (10 µM) or SEL120 (1µM) compared to vehicle-treated control (DMSO) cells (*n* = 4). Unpaired Student’s *t*-test was used to determine significance. * *ρ* < 0.05, ** *ρ* < 0.01, **** *ρ* < 0.0001.

**Figure 2:**
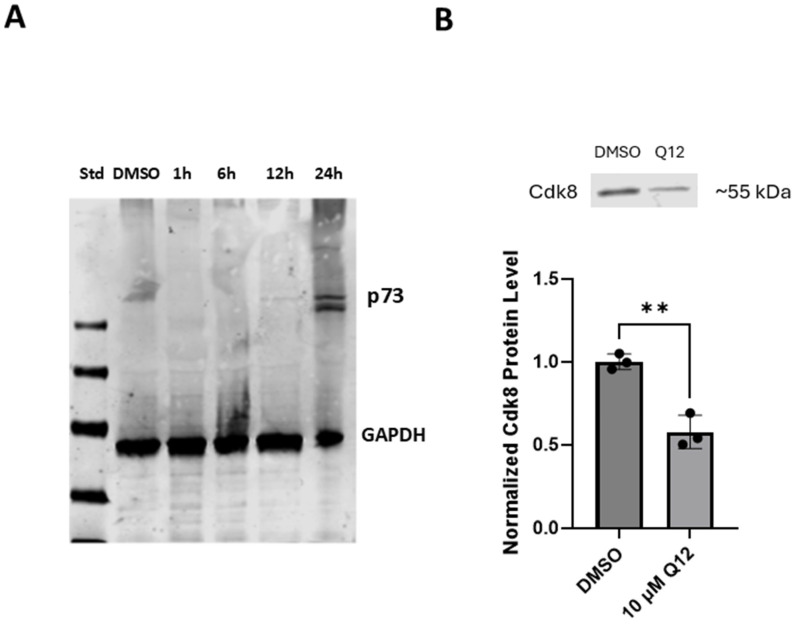
(**A**) p73 protein expression following treatment with 10 µM Q12 (1, 6, 12, 24 h) compared to vehicle-treated (DMSO) control (*n* = 3). (**B**) CDK8 protein expression in MDA-MB-468 cell line following 24 h treatment with 10 µM Q12 (*n* = 3). Images are representative of three independent experiments. Error bars represent mean ± SD. Unpaired Student’s *t*-test was used to determine significance. ** *ρ* < 0.01.

The E2F1 transcription factor is a phosphorylation substrate of CDK8 [6]. The CDK8 protein phosphorylates E2F1 at serine 375, and this phosphorylation event negatively regulates E2F1 transcriptional activity. To examine the effects of inhibiting CDK8 on E2F1 protein phosphorylation status in MDA-MB-468 cells, we used a small-molecule CDK8 inhibitor and Western blot technique. We treated cells with the CDK8 inhibitor Q12 and probed for E2F1 and serine 375-phosphorylated E2F1 (pE2F1(S375)) over time. As expected, pE2F1(S375) exhibited a large decrease by 6 h post treatment and was maintained for 24 h (Figure 1A). Total E2F1 protein had a small but significant decrease at 6h and continued to decrease over time. Using E2F1-responsive luciferase plasmid-transfected MDA-MB-468 cells, transcriptional activity of E2F1 was also assessed upon Q12 treatment compared to vehicle-treated control cells and cells treated with commercial CDK8 inhibitor SEL-120. Cells treated with CDK8 inhibitors Q12 and SEL-120 saw increased luciferase activity, whereas vehicle-treated control cells did not (Figure 1B). This result suggests that CDK8 suppresses E2F1 transcriptional activity in MDA-MB-468 cells. Additionally, we examined the effect of CDK8 inhibitor Q12 on E2F1 proapoptotic transcriptional target p73 [20,21]. The expression of p73 protein was noticeably increased by 24 h after Q12 treatment (Figure 2A).

**Figure 3:**
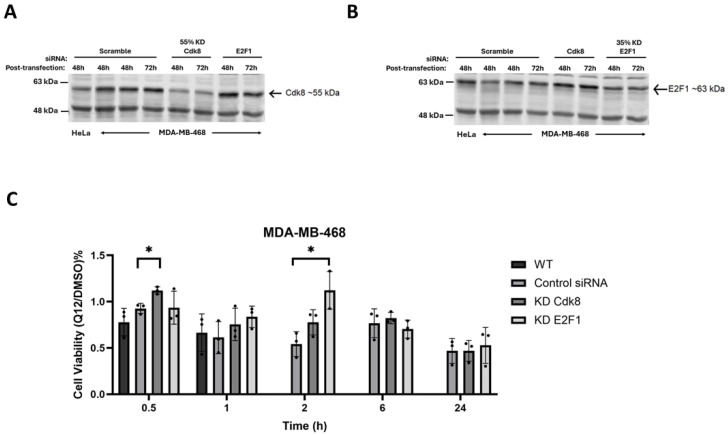
(**A**) Western blot assessment of siRNA-mediated CDK8 KD. (**B**) Western blot assessment of siRNA-mediated E2F1 KD. (**C**) Comparison of cell viability of wild-type, siRNA control-transfected, CDK8 siRNA-transfected, and E2F1 siRNA-transfected MDA-MB-468 cells treated with Q12 (20 µM) for 0.5, 1, 2, 6, and 24 h (*n* = 3). Only significant differences are shown. * *ρ* ≤ 0.01.

To determine whether the activation of E2F1-dependent transcription is critical for CDK8 inhibitor effects, we assessed cells transfected with siRNA against CDK8 and E2F1, then subsequently treated with a high concentration of Q12 for various lengths of time. CDK8 knockdown was approximately fifty-five percent efficient and E2F1 knockdown was approximately thirty-five percent efficient. CDK8 knockdown cells treated with Q12 had similar viability compared to control cells treated with Q12 at all timepoints tested as expected for complementary inhibition/depletion of CDK8. E2F1 knockdown seemed to have a protective effect at an early timepoint, but this effect was temporary as longer timepoints showed no difference, though this could also be due to low knockdown efficiency (Figure 3).

We also examined the effect of CDK8 inhibitor treatment of MDA-MB-468 cells on the CDK8 protein. Treatment of MDA-MB-468 cells with CDK8 inhibitor Q12 for 24 h resulted in a decrease in CDK8 protein (Figure 2B). This result demonstrates that CDK8 inhibitor Q12 not only inhibits the kinase activity of CDK8 but may also promote CDK8 protein degradation.

### 2.2. Effects of Inhibiting CDK8 on STAT3 and MDA-MB-468 Cell Viability

**Figure 4:**
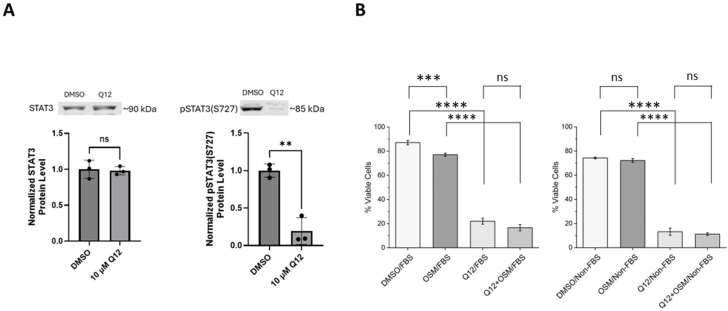
Effect of Q12 treatment on STAT3 phosphorylation status and cell viability in MDA-MB-468 cells grown in complete and in non-FBS-supplemented media. (**A**) STAT3 and pSTAT3 (Ser727) expression in MDA-MB-468 cell line after 24 h, 10 µM Q12 treatment compared to vehicle-treated control (DMSO) (*n* = 3). (**B**) Effect of treatment with 10 nM OSM, 10 µM Q12, or 10 nM OSM + 10 µM Q12 (72 h) on cell viability of MDA-MB-468 (MDA) cells compared to vehicle-treated control (DMSO) cells in complete media and serum-free media (*n* = 3). Images are representative of three independent experiments. Error bars represent mean ± SD. Unpaired Student’s *t*-test was used to determine significance. ** *ρ* < 0.01, *** *ρ* < 0.001, **** *ρ* < 0.0001, ns = not significant.

The STAT3 transcription factor is a phosphorylation substrate of CDK8 [22]. The CDK8 protein phosphorylates STAT3 on serine 727 (pSTAT3(S727)). Furthermore, the phosphorylation status of STAT3 serine 727 affects the phosphorylation status of the nearby tyrosine 705 [23]. We examined the phosphorylation status of STAT3 at serine 727 in MDA-MB-468 cells treated with CDK8 inhibitor Q12. Phosphorylation at serine 727 of STAT3 decreased compared to non-treated control cells (Figure 4A). This result is consistent with Q12 inhibiting the kinase function of CDK8. Treatment with Q12 did not affect total STAT3 levels. Next, we examined the ability of Q12 to decrease cell viability of MDA-MB-468 cells grown in FBS-supplemented media and non-FBS-supplemented media. Oncostatin M (OSM) treatment was included as a STAT3-activating treatment for comparison. Treatment with the CDK8 inhibitor Q12 with or without FBS or OSM supplementation dramatically reduced cell viability of MDA-MB-468 cells (Figure 4B). Treatment with OSM alone has little effect on cell viability. This result suggests that cytokine-induced activation of STAT3 is not necessary for the reduction in MDA-MB-468 cell viability caused by CDK8 inhibitor Q12.

### 2.3. Effect of Inhibiting CDK8 and STAT3 Knockdown on Mcl-1 Protein

The Mcl-1 protein is an antiapoptotic member of the Bcl-2 family [24]. Both E2F1 and STAT3 transcription factors are known to modulate Mcl-1 protein levels [25,26].

**Figure 5:**
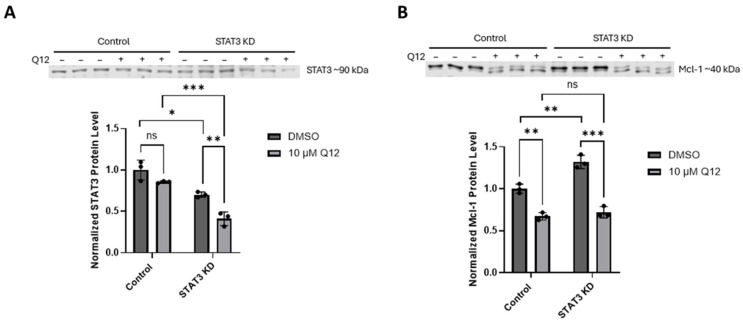
(**A**) Effect of siRNA-induced STAT3 knockdown (24 h) in MDA-MB-468 cells on STAT3 protein (upper) compared to cells treated with control siRNA (control) and cells treated with Q12 (10 µM, 24 h) and vehicle-treated (DMSO) control cells. (**B**) Effect of STAT3 knockdown on Mcl-1 protein (lower) in MDA-MB-468 cells compared to control cells treated with Q12 (10 µM, 24 h) and vehicle-treated (DMSO) control cells (*n* = 3). Error bars represent mean ± SD. Unpaired Student’s *t*-test was used to determine significance. * *ρ* < 0.05, ** *ρ* < 0.01, *** *ρ* < 0.001, ns = not significant.

To determine whether Mcl-1 protein is affected in MDA-MB-468 cells by treatment with CDK8 inhibitor Q12, we evaluated the effect of Q12 treatment on Mcl-1 and total STAT3 levels in MDA-MB-468 cells, as well as the effect of STAT3 knockdown on Mcl-1 protein level in MDA-MB-468 cells. We found that Q12 treatment reduced antiapoptotic protein Mcl-1, but did not significantly affect STAT3 protein level. Using RNA interference (siRNA), we were able to knockdown STAT3 protein by thirty percent, and even this relatively small decrease resulted in a significant increase in Mcl-1 protein compared to control siRNA-treated cells (Figure 5). Q12 treatment similarly reduced Mcl-1 protein in both control siRNA-treated cells and STAT3 siRNA-treated cells. These results support a hypothesis that the reduced cell viability and induction of apoptosis in MDA-MB-468 cells when treated with CDK8 inhibitor Q12 is due to modulation of antiapoptotic protein Mcl-1.

**Figure 6:**
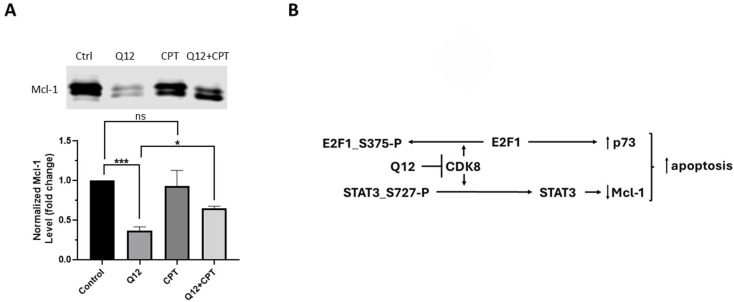
(**A**) Expression of Mcl-1 protein in MDA-MB-468 cells treated with Q12 (10 µM, 24 h), cryptotanshinone (CPT, 10 µM, 24 h), or co-treatment with Q12 (10 µM, 24 h) and CPT (10 µM, 24 h) compared to vehicle-treated (DMSO) control cells (*n* = 3). (**B**) Proposed mechanism by which CDK8 inhibitor decreases viability of MDA-MB-468 triple-negative breast cancer cells. Images are representative of three independent experiments. Error bars represent mean ± SD. Unpaired Student’s *t*-test was used to determine significance. * *ρ* < 0.05, *** *ρ* < 0.001, ns = not significant.

Previously, we demonstrated that co-treatment of MDA-MB-468 cells with STAT3-disrupter cryptotanshinone and Q12 attenuated the decrease in cell viability induced by Q12 treatment alone, and this is consistent with the ability of STAT3 to suppress Mcl-1 expression because of Q12 treatment. We next determined the effects of co-treatment with STAT3-disrupter cryptotanshinone and Q12 compared to cryptotanshinone or Q12 alone on Mcl-1 protein in MDA-MB-486 cells. Treatment with Q12 suppressed Mcl-1 protein levels and co-treatment of Q12 with cryptotanshinone attenuated the ability of Q12 to suppress Mcl-1 levels (Figure 6A). Cryptotanshinone alone did not affect Mcl-1 protein levels. These results are consistent with our previous studies, demonstrating the importance of STAT3 in the maintenance of Mcl-1 levels and providing a mechanism by which CDK8 inhibition reduces cell viability and induces apoptosis in MDA-MB-468 cells.

## 3. Discussion

The triple-negative breast cancer cell line MDA-MB-468 exhibits genomic alterations including overexpression of EGFR, mutant p53, and PTEN proteins, and does not express functional retinoblastoma (Rb) protein [27]. One of the functions of Rb protein is to sequester, through protein–protein interaction, the transcription factor E2F1 and prevent its function [28]. This interaction also protects E2F1 from ubiquitination and subsequent degradation [29]. The overexpression of E2F1 in MDA-MB-468 and other Rb-deficient cell lines induces apoptosis, indicating that negative regulation of E2F1, other than by Rb, exists in this cell line, preventing E2F1-induced apoptosis under normal circumstances [16]. The kinase function of CDK8 phosphorylates E2F1 at serine 375 and inhibits E2F1 transcriptional activity [6]. We hypothesized that the CDK8 protein may be an important regulator of E2F1 in this cell line and that, together with lack of protection by absent Rb, E2F1 function is kept low and that this may help the cells evade apoptosis. Previously, we demonstrated that treatment of MDA-MB-468 cells with CDK8 inhibitor Q12 reduced cell viability and induced apoptosis [17]. Here, we show that treatment with Q12 causes a decrease in CDK8 protein and a decrease in pE2F1(S375). We demonstrated, by use of an E2F1-dependent luciferase assay, that inhibition of CDK8 by Q12 induced E2F1 transcriptional activity. Additionally, we examined the effect of CDK8 inhibitor Q12 on E2F1 transcriptional target p73. The expression of proapoptotic p73 protein increases noticeably by 24 h after Q12 treatment. This result is consistent with the hypothesis that CDK8 inhibition can induce apoptosis in MDA-MB-468 cells by reactivation of E2F1 transcriptional activity that is normally silent because of phosphorylation by CDK8. This is supported by the delayed effects on cell viability of Q12-treated cells after partial knockdown of E2F1. E2F1 activity in triple-negative breast cancer patients is clinically relevant, as one study showed that patients that respond to doxorubicin treatment had upregulated E2F1 in tumor biopsies and non-responders did not [30]. Our previous report also demonstrated that CDK8 inhibitor Q12 increased pSTAT3(Y705), and we wondered if this phenomenon could contribute to induction of apoptosis in MDA-MB-468 cells.

The CDK8 protein phosphorylates STAT3 on serine 727, and this has a destabilizing effect on nearby tyrosine 705 phosphorylation [23]. Inhibition of CDK8 causes a decrease in pSTAT3(S727) and a corresponding increase in pSTAT3(Y705) as it becomes more stable. It has been shown that overstimulation of MDA-MB-468 cells with cytokines like epidermal growth factor (EGF) can induce apoptosis accompanied by increased STAT3 phosphorylation [31]. To determine if increased pSTAT3(Y705) contributes to the reduction in cell viability caused by Q12 treatment, we compared cell viability in both FBS- and non-FBS-supplemented media or treatment with cytokine OSM. Treatment of MDA-MB-468 cells with Q12 reduced cell viability in FBS-, OSM-, and non-FBS-supplemented media suggesting that increased STAT3 activation by cytokines is not necessary for, but may contribute to, the cell-viability-reducing effect of Q12.

Both E2F1 and STAT3 can transcriptionally modulate apoptosis-related proteins. We demonstrate that treatment of MDA-MB-468 cells with CDK8 inhibitor Q12 results in increased proapoptotic protein p73 and decrease in antiapoptotic protein Mcl-1. This is consistent with increased E2F1 activity that promotes transcription of p73 and E2F1 antagonistic effect on Mcl-1 expression. We also demonstrate that knockdown of STAT3 protein in MDA-MB-468 results in an increase in Mcl-1 protein and is consistent with our previous report that co-treatment of MDA-MB-468 cells with STAT3-disruptor cryptotanshinone and Q12 attenuated the effects of Q12 on cell viability. Additionally, here, we demonstrate that co-treatment of MDA-MB-468 cells with crytotanshinone and Q12 attenuates Q12 suppressive effects on Mcl-1. In other words, the ability of cryptotanshinone to prevent dimerization and DNA binding of STAT3 mirrors STAT3 knockdown [32]. This suggests that STAT3, in one form or another, has a negative regulatory effect on Mcl-1, and that Mcl-1 can promote MDA-MB-468 cell survival. It has been reported that phosphorylation of STAT3 at Ser727 negatively regulates transcriptional activity by decreasing promoter residence time and increasing nuclear export [22]. Inhibition of CDK8 may reverse this effect and suggests a suppressive role for STAT3 upon the Mcl-1 gene in the MDA-MB-468 cell line. It is an intriguing possibility that the presence of STAT3 keeps Mcl-1 at a basal level that contributes to a ‘primed for death’ state of the cell in which further inhibition of Mcl-1 initiates apoptosis [33]. Further inhibition of Mcl-1 can be accomplished by CDK8 inhibitor-initiated activation of E2F1 and/or enhanced STAT3-mediated suppression of Mcl-1. In other words, dephosphorylation of STAT3(S727) by CDK8 inhibitor may enhance STAT3 suppression of Mcl-1, whereas knockdown of, or disruption of, STAT3 by cryptotanshonone may prevent STAT3 suppression of Mcl-1. Additionally, CDK8 inhibitor-reactivated E2F1 promotes the expression of proapoptotic protein p73. Significantly, Mcl-1 antagonizes the proapoptotic effects of p73 through direct binding of the p73 BH3-like domain [34]. Figure 6B represents a proposed mechanism by which CDK8 inhibitor Q12 decreases cell viability of MDA-MB-468 triple-negative breast cancer cells.

In summary, we have demonstrated some of the effects of CDK8 inhibition on CDK8, E2F1, STAT3, and Mcl-1 proteins in MDA-MB-468 triple-negative breast cancer cells. We have also revealed that partial knockdown of STAT3 in MDA-MB-468 cells can result in an increase in Mcl-1 protein expression. The potential additive or synergistic effects between increased E2F1 activity and STAT3 suppressive activity on Mcl-1 due to CDK8 inhibition in MDA-MB-468 cells is intriguing. These results suggest that Mcl-1 is a critical protein for MDA-MB-468 cell survival. This suggestion is corroborated by a recent report demonstrating that treatment with a small-molecule Mcl-1-selective inhibitor induces apoptosis in MDA-MB-468 cells [35]. Inhibiting the kinase function of CDK8 modulates the activity of two transcription factors, E2F1 and STAT3, toward a cooperative effect, resulting in cell death. Currently, both CDK8 and Mcl-1 are being actively pursued as therapeutic targets in breast cancer in general and triple-negative breast cancer specifically [2,15,19,35,36,37,38,39,40,41,42]. We believe that the data and context presented here will stimulate more interest in CDK8 and its interacting partners as potential therapeutic drug targets and biomarkers. We continue to investigate the contributions of E2F1 and STAT3 to CDK8 inhibitor effects on triple-negative breast cancer cell line MDA-MB-468, and will report our results in due course.

## 4. Materials and Methods

### 4.1. Cell Lines and Reagents

MDA-MB-468 cells were obtained from the American Type Culture Collection, Manassas, VA, USA (ATCC, #HTB-132) and maintained in Advanced MEM (Gibco, Gaithersburg, MD, USA) supplemented with 5% FBS (Cytiva, Logan, UT, USA), streptomycin (100 μg/mL) and penicillin (100 units/mL) (Gibco), and 1% GlutaMAX (Gibco). Cells were maintained as a monolayer at 37 °C and 5% CO_2_. Control siRNA-B (sc-44230), E2F-1 siRNA (h) (sc-29297), Cdk8 siRNA (h) (sc-29267), siRNA Transfection Medium (sc-36868) and siRNA Transfection Reagent (sc-29528), and mouse anti-Mcl-1 (22, sc-12756) (dil. 1:400) were purchased from Santa Cruz Biotechnology, Inc. (Santa Cruz, CA, USA). Rabbit anti-E2F-1 (#3742S (dil. 1:1000)), anti-Cdk8 (G398, #4101S) (dil. 1:1000), anti-STAT3 (#30835S) (dil. 1:1000), anti-pSTAT3(S727) (#9134S) (dil. 1:1000), anti-p73 (#14620) (dil. 1:1000), and anti-GAPDH (4C10, #5174S) (dil. 1:5000) antibodies were purchased from Cell Signaling Technology (Danvers, MA, USA). Rabbit anti-pE2F1(S375) (#MABE1782) antibody was purchased from Millipore-Sigma (St. Louis, MO, USA) (dil. 1:1000). Secondary donkey antibody conjugated with IRDye 800CW was purchased from LI-COR Bioscience (Lincoln, NE, USA) (dil. 1:10,000). CellTiter 96^®^ AQueous One Solution Cell Proliferation Assay (MTS) was purchased from Promega (Madison, WI, USA). Q12 was synthesized and purified according to the published procedure [18]. SEL120 was purchased from SelleckChem (SEL120-34A) (Selleck Chemicals, LLC, Houston, TX, USA). pGL2-AN was a gift from William Kaelin (Addgene plasmid # 20950; http://n2t.net/addgene:20950 (accessed on 25 April 2023); RRID:Addgene_20950) [43]. For experiments where Q12 was used, a 10 µM concentration was applied for 24 h or less; no visual signs of cell death were noted. In cell viability experiments where treatment times were 72 h or when Q12 was used at 20 µM, an increased fraction of non-adherent cells was noted.

### 4.2. Dual-Glo Luciferase Assay

MDA-MB-468 cells were seeded in a 96-well plate reaching 90–95% confluency at the time of transfection. Plasmid DNA, EndoFectin™ transfection reagent (GeneCopoeia EF014, Rockville, MD, USA), and Opti-MEM (Gibco, 31985-062) were equilibrated to room temperature before use. Cells were co-transfected with 200 ng of plasmids (100 ng of pGL2-AN-E2F1 luciferase reporter construct and 100 ng of pRL-TK Renilla luciferase plasmid) and 0.4 μL of transfection reagent for each well. Both the plasmids and the transfection reagent were diluted in the Opti-MEM, respectively. Then, the diluted transfection reagent and the diluted DNA were combined and kept at room temperature for 20 min to allow DNA-transfection reagent complexes to form. The combined complexes were added to each well and mixed gently. The cells were harvested for analysis after 48 h of incubation at 37 °C, 5% CO_2_. Treatment of 0.1% DMSO, 10 μM Q12, or 1 μM SEL120 was added at 24 h post transfection. E2F1-induced transcriptional activity was determined according to Dual-Glo^®^ Luciferase Assay System Protocol (Promega, Madison, WI, USA). Briefly, the 96-well plate containing 75 μL of culture medium in each well was taken out from the incubator. Then, 75 μL of Dual-Glo^®^ Reagent was added to each well and mixed well. After 10 min, the firefly luminescence was measured in GloMax^®^ Discover Microplate Reader (Promega, Madison, WI, USA). 75 μL of Dual-Glo^®^ Stop & Glo^®^ Reagent was added to each well and mixed well. After 10 min, the Renilla luminescence was measured in the same plate order as the firefly luminescence. Relative luciferase activity was represented as a ratio of firefly luminescence/Renilla luminescence, which was normalized to the ratio of the DMSO-treated well (control group).

### 4.3. Cell Viability Assay

Cells were treated with DMSO (0.1%, vehicle control), 10 µM Q12, 10 nM OSM (Thermo Fisher Science, San Jose, CA, USA) or 10 µM Q12 and 10 nM OSM for 72 h. Floating cells and adherent cells were collected and combined the various treatments. Cells were washed twice with 1× PBS and re-suspended in fresh media for cell viability assay. Cell viability was measured with the Muse Cell Analyzer (Millipore, Hayward, CA, USA) using the Muse Count and Viability Kit (Millipore, Hayward, CA, USA).

### 4.4. Western Blot Analysis

In brief, whole-cell lysates were prepared by three cycles of freeze/thaw and then incubation on ice for 30 min in HEDG buffer (25 mM HEPES, pH 7.4, 1 mM EDTA, 1 mM DTT, 10% glycerol) containing 0.4 M KCl, 1 mM PMSF, and 2 µg/mL of leupeptin, followed by centrifugation (16,000× *g* for 15 min at 4 °C). BCA protein assay (Thermal Fisher, Waltham, MA, USA) was used to determine the protein content. Typically, 30 μg of lysates were used for Western blot analysis and separated by 12% SDS-PAGE. Wet transfer was performed to transfer proteins from gel to nitrocellulose membrane at 300 mA for 120 min at 4 °C. Membrane was incubated for 1 h at room temperature in blocking solution (PBS containing 5% BSA, 0.1% Tween-20, and 0.05% sodium azide), followed by primary-antibody incubation overnight at 4 °C. Incubation with secondary donkey antibody conjugated with IRDye was carried out in blocking solution for 2 h at room temperature. A washing step (5 repeats of 5 min wash with PBS + 0.1% Tween-20) was performed after antibody incubation. The results were analyzed using a LI-COR CLx Odyssey imager (LI-COR Biotech, Lincoln, NE, USA). The amount of target proteins was normalized with the total protein using the Revert total protein stain (LI-COR) or by comparison to GAPDH. In general, cells used for Western blot analysis were treated with DMSO (0.1%, vehicle control), Q12 (10 µM), cryptotanshinone (10 µM), or combined unless otherwise indicated. Treatment exposure was for 24 h unless otherwise indicated.

### 4.5. Transient siRNA Transfection

Transient transfection was performed using the siRNA transfection reagent (Santa Cruz Biotechnology, Santa Cruz, CA, USA) following the manufacturer’s instructions. Briefly, MDA-MB-468 cells were plated at a density of 2 × 10^5^ cells per well of a 6-well plate and cultured in 2 mL of antibiotic-free Advanced MEM medium supplemented with 5% FBS and 1% GlutaMAX. Six micrograms of a siRNA duplex was delivered into cells with six microliters of siRNA transfection reagent. Cells were harvested or treated 48 h after transfection.

### 4.6. Cell Viability Assay of siRNA-Transfected Cells

MDA-MB-468 cells were plated in 96-well plates at a density of 1.25 × 10^4^ per well and transiently transfected following the protocol mentioned above, scaled down. Cells were treated with either DMSO or Q12 (20 µM) 48 h after transfection. CellTiter 96^®^ AQueous One Solution Cell Proliferation Assay (MTS) was added to cells at 0.5, 1, 2, 6, and 24 h after treatment. Cell viability was analyzed using GloMax^®^ Discover Microplate Reader and values were normalized by DMSO-treated samples.

### 4.7. Statistical Analysis

Data was analyzed using GraphPad Prism (Version 10.0.0 for Windows). Statistical analysis employed one-way or two-way ANOVA or Student’s *t* test. The number of asterisks in figures indicates significance as follows: *ρ* > 0.05 (ns, not significant), *ρ* < 0.05 (*), *ρ* < 0.01 (**), *ρ* < 0.001 (***), and *ρ* < 0.0001 (****).

## Data Availability

The original contributions presented in this study are included in the article. Further inquiries can be directed to the corresponding author.

## Abbreviations

The following abbreviations are used in this manuscript:

CDK8	Cyclin-Dependent Kinase 8
CPT	Cryptotanshinone
DMSO	Dimethylsulfoxide
E2F1	E2F Transcription Factor 1
FBS	Fetal Bovine Serum
GAPDH	Glyceraldehyde-3-phosphate Dehydrogenase
Mcl-1	Myeloid Cell Leukemia-1
MED13	Mediator Complex Subunit 13
OSM	Oncostatin M
Rb	Retinoblastoma
RNAPII	RNA Polymerase II
siRNA	Small Interfering RNA
STAT3	Signal Transducer and Activator of Transcription 3
TNBC	Triple-Negative Breast Cancer
